# Potential window alignment regulating ion transfer in faradaic junctions for efficient photoelectrocatalysis

**DOI:** 10.1038/s41467-023-43916-6

**Published:** 2023-12-02

**Authors:** Hongzheng Dong, Xiangyu Pan, Yuancai Gong, Mengfan Xue, Pin Wang, SocMan Ho-Kimura, Yingfang Yao, Hao Xin, Wenjun Luo, Zhigang Zou

**Affiliations:** 1 Eco-materials and Renewable Energy Research Center (ERERC), National Laboratory of Solid State Microstructures, College of Engineering and Applied Sciences, Nanjing University, Nanjing, 210093 China; 2 State Key Laboratory for Organic Electronics and Information Displays, College of Chemistry and Life Sciences, Nanjing University of Posts & Telecommunications, Nanjing, 210023 China; 3 Eco-materials and Renewable Energy Research Center (ERERC), Jiangsu Key Laboratory for Nano Technology, National Laboratory of Solid State Microstructures and Department of Physics, Nanjing University, Nanjing, 210093 China; 4 Institute of Applied Physics and Materials Engineering, University of Macau, Macau SAR, China

**Keywords:** Photocatalysis, Solar fuels

## Abstract

In the past decades, a band alignment theory has become a basis for designing different high-performance semiconductor devices, such as photocatalysis, photoelectrocatalysis, photoelectrostorage and third-generation photovoltaics. Recently, a faradaic junction model (coupled electron and ion transfer) has been proposed to explain charge transfer phenomena in these semiconductor heterojunctions. However, the classic band alignment theory cannot explain coupled electron and ion transfer processes because it only regulates electron transfer. Therefore, it is very significant to explore a suitable design concept for regulating coupled electron and ion transfer in order to improve the performance of semiconductor heterojunctions. Herein, we propose a potential window alignment theory for regulating ion transfer and remarkably improving the photoelectrocatalytic performance of a MoS_2_/Cd-Cu_2_ZnSnS_4_ heterojunction photocathode. Moreover, we find that a faradaic potential window, rather than the band position of the intermediate layer, is a criterion for identifying interface charge transfer direction. This finding can offer different perspectives for designing high-performance semiconductor heterojunctions with suitable potential windows for solar energy conversion and storage.

## Abstract (web summary)

Band alignment theory has been widely used to design semiconductor heterojunctions for photo(electro)catalysis, but it cannot describe coupled electron and ion transfer process. Here, the authors propose potential window alignment theory to remarkably improve the performance of heterojunctions.

## Introduction

In the past several decades, photocatalysis, photoelectrocatalysis, photoelectrostorage, third-generation photovoltaics (such as quantum-dot or dye-sensitized, organic and perovskite solar cells) have attracted wide interest due to their low cost and high efficiency for converting solar energy into chemical or electric energy^1–5^. Among them, different kinds of heterojunctions, such as type-II heterojunction^6^ and *Z*-scheme junction^7^, have been widely used to separate photo-excited carriers in light absorbers (Supplementary Fig. 1). In all of these previous studies^6–8^, a band alignment theory is a basis for manipulating interface electron transfer in high-performance devices. However, Luo et al. have found that faradaic junction interface charge transfer process (coupled electron and ion transfer) widely exists in these solar energy conversion and storage devices^9–17^. Since the classic band alignment theory only regulates electron transfer, it is not suitable for explaining coupled electron and ion transfer in faradaic junctions. Therefore, it is very significant to explore a suitable design concept for regulating coupled electron and ion transfer in order to improve the performance of the semiconductor heterojunctions.

In this work, we have improved the photoelectrochemical hydrogen production performance of a MoS_2_/Cd-Cu_2_ZnSnS_4_(CCZTS) heterojunction photocathode significantly by introducing an Au intermediate layer. However, a Pd intermediate layer with a similar Fermi level does not improve the performance of the same MoS_2_/CCZTS heterojunction photocathode. The results suggest that the classic band alignment theory is not suitable for explaining the different effects of the Pd and Au layers on the photoelectrochemical properties of CCZTS. In order to investigate the reasons for the improved performance of MoS_2_/Au/CCZTS heterojunction photocathode, some characterization methods, including in situ irradiated X-ray photoelectron spectra (XPS), quasi in situ irradiated Raman, in situ electrochemical Raman and time-of-flight secondary-ion mass spectrometry (TOF-SIMS), were carried out. We find that the MoS_2_ plays the role of recombination centers by a revisable faradaic reaction and decreases the performance of a MoS_2_/CCZTS heterojunction photocathode. The introduction of the Au intermediate layer inhibits alkali metal ion intercalation reaction of MoS_2_ and decreases the recombination in the MoS_2_/CCZTS heterojunction, but the Pd intermediate layer does not. Further studies suggest the Au layer indicates negligible reduction faradaic potential window, compared to the Pd layer, which suppresses alkali metal ion intercalation into MoS_2_. Therefore, the theory of the potential window alignment can be used to explain the interface charge transfer in photoelectrocatalysis and can also provide guidance to improve the performance of photocatalysis, photoelectrostorage, third-generation photovoltaics and so on.

## Results

### The Au intermediate layer to improve photoelectrocatalytic performance of a MoS_2_/CCZTS heterojunction photocathode remarkably

**Fig. 1:**
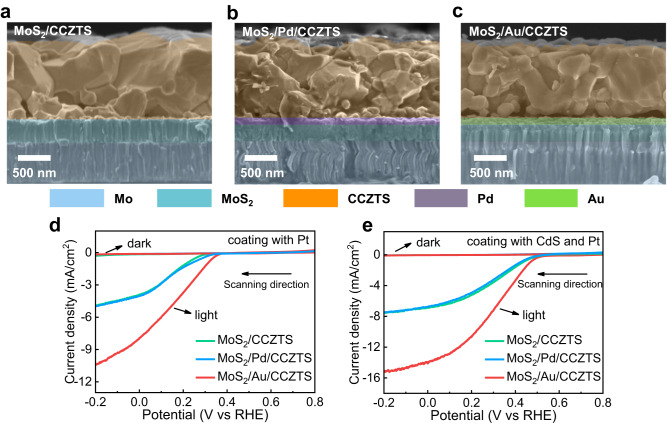
Characterization and photocurrent of MoS_2_/CCZTS, MoS_2_/Pd/CCZTS and MoS_2_/Au/CCZTS samples. **a**–**c** The cross-sectional SEM images of MoS_2_/CCZTS (**a**), MoS_2_/Pd/CCZTS (**b**) and MoS_2_/Au/CCZTS (**c**). **d**, **e** Linear sweep voltammetry curves of MoS_2_/CCZTS, MoS_2_/Pd/CCZTS and MoS_2_/Au/CCZTS after coating a Pt co-catalyst (**d**) or coating a CdS buffer layer and a Pt co-catalyst (**e**); Scan rate of 10 mV/s; Electrolyte: 0.2 M Na_2_HPO_4_/NaH_2_PO_4_ aqueous solution (pH = 6.5). Light source: an AM 1.5 G sunlight simulator, light intensity: 100 mW/cm^2^.

In previous studies, Mo glass was widely used as a substrate for the preparation of a CCZTS thin film due to its good mechanical strength at high temperature^18,19^. Here, CCZTS films were deposited on Mo glass substrates by spin-coating and post-sulfurization method. After the sulfurization at high temperature, MoS_2_ was inevitably in situ produced on the surface of Mo glass substrates, which contacts with CCZTS to form MoS_2_/CCZTS heterojunction^20,21^. For MoS_2_/Pd/CCZTS and MoS_2_/Au/CCZTS samples, Pd and Au intermediate layers were deposited firstly on Mo glass substrates with an ion beam sputtering method and then CCZTS layers were prepared. The preparation details of the three samples can be found in the Methods section. The three samples were characterized by XRD (Supplementary Fig. 2a). The diffraction peaks at 28.4°, 47.3° and 56.2° correspond to the (112), (220) and (312) planes of kesterite CCZTS^22,23^, and the peaks of Au can also be observed in MoS_2_/Au/CCZTS sample. However, no diffraction peaks of Pd and MoS_2_ are observed in the three samples by the conventional XRD method due to their poor crystallinity. In order to further identify the Pd and MoS_2_, the CCZTS layers were removed by a tweezer and Grazing Incidence X-Ray Diffraction (GIXRD) was used to characterize the exposed MoS_2_ and MoS_2_/Pd substrates (Supplementary Fig. 2b). By this method, the diffraction peaks of Pd and 2H-MoS_2_ are observed. Figure 1a–c show the cross-sectional SEM images of MoS_2_/CCZTS, MoS_2_/Pd/CCZTS and MoS_2_/Au/CCZTS, respectively. In the three samples, well-grown CCZTS grains with different sizes are packed on the substrates. Therefore, the Pd and Au intermediate layers do not change the morphologies of CCZTS obviously. Moreover, both Pd and Au intermediate layers between MoS_2_ and CCZTS indicate small particle aggregates, which are about 80 nm in thickness (Supplementary Figs. 3, 4) and cover the MoS_2_ underlayers incompletely in the heterojunctions from the top-view SEM images by removing the upper layers of CCZTS (Supplementary Fig. 5).

In order to investigate the effects of Pd and Au intermediate layers on the photoelectrochemical performance of CCZTS-based photocathodes, the photocurrent density of MoS_2_/CCZTS, MoS_2_/Pd/CCZTS and MoS_2_/Au/CCZTS samples were measured in 0.2 M Na_2_HPO_4_/NaH_2_PO_4_ aqueous solution. From previous studies^24^, the photocurrent of the bare CCZTS sample is negligible due to its low electrocatalytic activity for hydrogen production. Therefore, Pt co-catalysts were loaded on the surfaces of the three samples by an ion beam sputtering method for photoelectrocatalytic measurement. Figure 1d indicates Linear sweep voltammetry (LSV) curves of the three samples with Pt cocatalysts. Usually, a more negative potential has a larger driving force for electron-hole separation in a photocathode, which will lead to a higher photocurrent at a more negative potential. Therefore, the photocurrent of all of the three samples increases with shifting the potential negatively. It is worth noting that the photocurrent of CCZTS increases significantly after introducing the Au layer. In contrast, the MoS_2_/Pd/CCZTS exhibits a similar photocurrent to that of the MoS_2_/CCZTS photocathode. Moreover, n-CdS was also introduced as a buffer layer between CCZTS and Pt co-catalysts to further improve the photoelectrocatalytic performance^22,23^. The photocurrent density of MoS_2_/CCZTS increases from 7.2 mA/cm^2^ to 14.9 mA/cm^2^ @ 0 V_RHE_ after introducing the Au intermediate layer, but slightly decreases after introducing the Pd layer (Fig. 1e), which is further confirmed by the statistical results in the Supplementary Fig. 6. Moreover, the three samples also indicate good stability under illumination and the photocurrents only decay by about 5–11% after 3600 s of illumination (Supplementary Fig. 7). Therefore, the Au intermediate layer can improve the photoelectrocatalytic performance of the MoS_2_/CCZTS heterojunction photocathode remarkably.

**Fig. 2:**
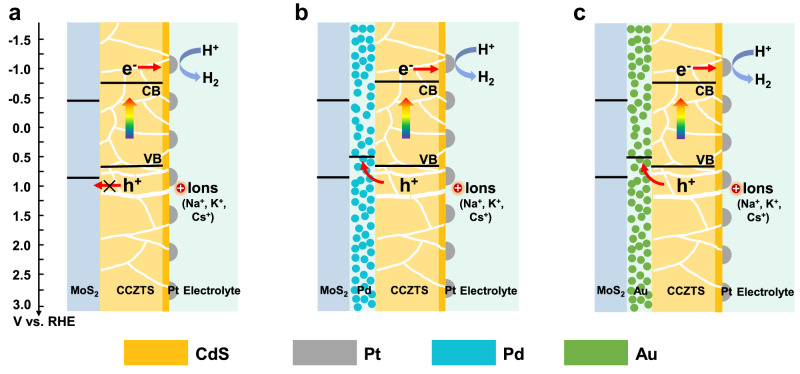
Conventional band alignment theory for interface charge transfer. **a**–**c** The diagram of interface charge transfer in MoS_2_/CCZTS (**a**), MoS_2_/Pd/CCZTS (**b**) and MoS_2_/Au/CCZTS (**c**) heterojunction photocathodes.

In order to investigate the reason that enhanced photocurrent is only observed in MoS_2_/Au/CCZTS heterojunction photocathode, but not in MoS_2_/Pd/CCZTS, we measured the substrate resistance and light absorption of the samples and the results are shown in Supplementary Table 1 and Supplementary Fig. 8, respectively. The MoS_2_/Au substrate exhibits similar resistance as the MoS_2_/Pd substrate and the light absorption of CCZTS does not change obviously on different substrates, suggesting that the difference in performance of these samples does not come from the change of the substrate resistance and light absorption. Since a band alignment theory is usually used to understand the improved performance in the heterojunction, the band gaps and band positions of four CCZTS and MoS_2_ samples were measured by UV-vis spectra and Mott-Schottky methods, respectively (Supplementary Figs. 9, 10). The average values and errors of the band gaps and band positions of the four samples are calculated and shown in Supplementary Table 2, which are in good agreement with some previous reports^23,25^. Moreover, the Fermi levels (E_F_) of Pd and Au particulate films in this study were obtained by ultraviolet photoelectron spectroscopy (Supplementary Fig. 11) and the values are summarized in Supplementary Table 3, which are similar to the reference values in previous studies^26,27^. According to the above values, the band positions of MoS_2_/CCZTS, MoS_2_/Pd/CCZTS and MoS_2_/Au/CCZTS heterojunction photocathodes are plotted in Fig. 2. Based on the band alignment theory, the valence band of CCZTS is higher than that of MoS_2_, unfavorable for photo-generated hole transferring from the CCZTS photocathode to MoS_2_. Therefore, the Pd or Au intermediate layers with the higher Fermi level will be helpful for hole transfer from CCZTS to the metal layers, which can improve the performance of a CCZTS photocathode^28^. However, in experiments, not the Pd but the Au intermediate layer improves the photocurrent for solar hydrogen production. Therefore, the classic band alignment theory is not suitable for explaining the converse effects of the Pd and Au layers on the photoelectrochemical properties of CCZTS.

### The Au intermediate layer to regulate ion transfer in the MoS_2_/CCZTS heterojunction photocathode under illumination

**Fig. 3:**
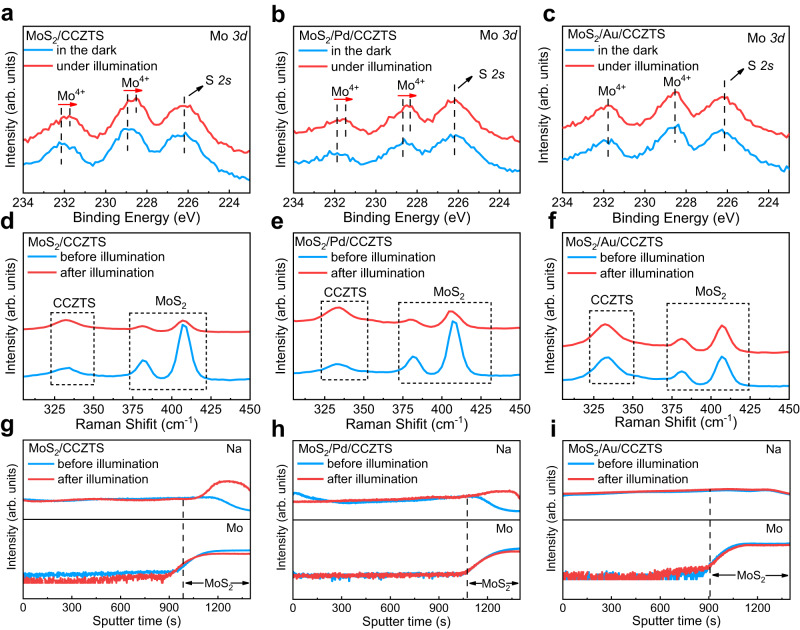
Interface charge transfer process in MoS_2_/CCZTS, MoS_2_/Pd/CCZTS and MoS_2_/Au/CCZTS samples. **a**–**c** In situ irradiated XPS spectra of Mo *3d* in MoS_2_/CCZTS (**a**), MoS_2_/Pd/CCZTS (**b**) and MoS_2_/Au/CCZTS (**c**) in the dark and under full arc Xe lamp illumination. The coating and annealing were repeated two times to obtain a detectable XPS signal of Mo. **d**–**f** Quasi in situ irradiated Raman spectra of MoS_2_/CCZTS (**d**), MoS_2_/Pd/CCZTS (**e**) and MoS_2_/Au/CCZTS (**f**) in the electrolyte before and after full arc Xe lamp illumination. **g**–**i** Secondary ion intensities of Na and Mo ions in MoS_2_/CCZTS (**g**), MoS_2_/Pd/CCZTS (**h**) and MoS_2_/Au/CCZTS (**i**) junctions in the electrolyte before and after full arc Xe lamp illumination. Electrolyte: 0.2 M Na_2_HPO_4_/NaH_2_PO_4_ aqueous solution; Light source: a full arc Xe lamp.

According to previous studies^9–16^, a faradaic junction charge transfer widely exists in different heterojunctions. Herein, we investigated the effects of Pd and Au on interface charge transfer in MoS_2_/CCZTS by in situ irradiated XPS and the experimental details are shown in Supplementary Fig. 12. Since XPS is a surface characterization technique, it is difficult to obtain the Mo signal in the substrate if the CCZTS upper layer is too thick and compact. A thinner and porous CCZTS upper layer was prepared by only reducing the number of coating layers from seven to two with the same method, and the XPS peak of Mo 3d becomes more intensive in the MoS_2_/CCZTS sample (Supplementary Fig. 13). Therefore, the MoS_2_/CCZTS sample with two coating layers was investigated by in situ irradiated XPS and the results are shown in Fig. 3a–c. Two binding energies at 231.9 eV and 228.8 eV are assigned as Mo^4+^ in the MoS_2_/CCZTS sample in the dark (Fig. 3a), which shift to lower values under illumination^29^. The results suggest that Mo^4+^ ions are photo-reduced into lower valence states^30^. After introducing Pd and Au intermediate layers, lower binding energies of Mo are only observed in MoS_2_/Pd/CCZTS, but not in MoS_2_/Au/CCZTS under illumination (Fig. 3b, c). The results suggest that the Au rather than Pd intermediate layer can prevent the reduction of Mo^4+^ in the MoS_2_/CCZTS junction. On the other hand, the binding energies of Cu *2p*, Zn *2p*, Sn *3d* and S *2p* in the three samples do not shift under illumination, suggesting that the CCZTS does not change (Supplementary Figs. 14–16). Quasi in situ irradiated Raman spectra were also used to investigate the effects of Au and Pd on the reduction process of MoS_2_ in MoS_2_/CCZTS before and after illumination^31^ and the experimental details are shown in Supplementary Fig. 17. The Raman peaks at 382 cm^−1^ and 408 cm^−1^ are assigned as *E*^*1*^_*2g*_ and *A*_*1g*_ vibrational modes of MoS_2_, and the peak at 332 cm^−1^ corresponds to CCZTS^22,29^. After MoS_2_/CCZTS is illuminated, the Raman peak intensity of CCZTS does not change, while the Raman peak intensity of MoS_2_ decreases (Fig. 3d). In contrast, the Raman peak intensity of MoS_2_ film alone does not decrease after the same illumination (Supplementary Fig. 18). Therefore, interface charge transfer in the MoS_2_/CCZTS heterojunction under illumination leads to the change of the Raman peak intensity of MoS_2_. According to previous studies^32,33^, the decrease of Raman peak intensity of MoS_2_ can be attributed to the Na^+^ intercalation into MoS_2_. The Raman peak intensity of MoS_2_ decreases in MoS_2_/Pd/CCZTS but not in MoS_2_/Au/CCZTS after illumination (Fig. 3e, f). The results further suggest that the Au rather than Pd intermediate layer can inhibit the reduction intercalation reaction of MoS_2_ in the MoS_2_/CCZTS junction, which are in good agreement with in situ irradiated XPS results mentioned above.

In order to further investigate the effects of Au and Pd on interface ion transfer in MoS_2_/CCZTS under illumination, time-of-flight secondary ion mass spectrometry (TOF-SIMS) depth profiles were used to identify the element distribution in the three samples in 0.2 M Na_2_HPO_4_/NaH_2_PO_4_ aqueous solution before and after illumination. Before illumination, Na^+^ ions uniformly distribute in the bulk of the CCZTS film and decrease at the MoS_2_/CCZTS interface. After illumination, the distribution of Cu, Zn, Sn and S in CCZTS does not change obviously (Supplementary Fig. 19), while the intensity of Na^+^ ions enhances remarkably at the depth of sputtering time >980 s in the sample (Fig. 3g), where the MoS_2_ is located. Since some pin holes inevitably exist even in the dense and thick CCZTS films prepared by the spin-coating method, electrolyte can penetrate into the MoS_2_ layer through the pin holes (Supplementary Fig. 20). Therefore, the Na^+^ intercalation into MoS_2_ happens in the MoS_2_/CCZTS sample in the electrolyte under illumination. According to in situ irradiated XPS and TOF-SIMS, the MoS_2_/CCZTS is a faradaic junction with the interface reaction of MoS_2_ + x Na^+^ + x e^−^ ↔ Na_x_MoS_2_. In the MoS_2_/Pd/CCZTS junction, similar Na^+^ intercalation into MoS_2_ was observed after illumination (Fig. 3h and Supplementary Fig. 21). In contrast, the distribution of Na^+^ in the MoS_2_/Au/CCZTS junction does not change after illumination (Fig. 3i and Supplementary Fig. 22). Therefore, the Na^+^ intercalation into MoS_2_ is suppressed only by the Au rather than Pd intermediate layer in MoS_2_/CCZTS.

**Fig. 4:**
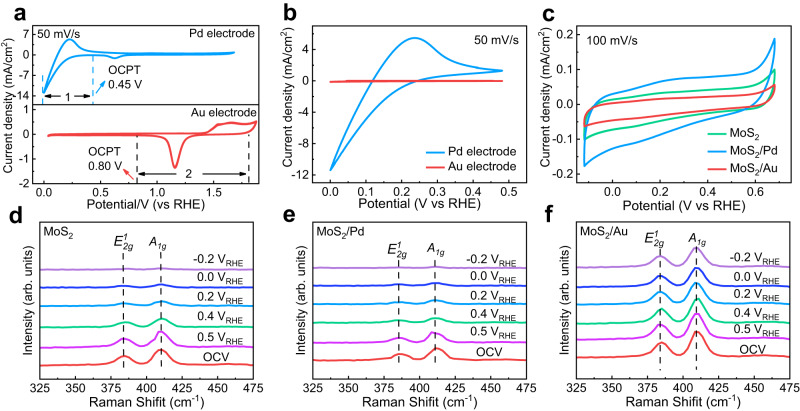
The reasons of Au rather than Pd to inhibit Na^+^ intercalation into MoS_2_. **a**–**c** Cyclic voltammetry curves of Pd and Au electrodes (**a**, **b**), MoS_2_, MoS_2_/Pd and MoS_2_/Au (**c**) in the electrolyte in the dark. **d**–**f** In situ electrochemical Raman spectra of MoS_2_ (**d**), MoS_2_/Pd (**e**) and MoS_2_/Au (**f**) in the electrolyte at different potentials in the dark. Electrolyte: 0.2 M Na_2_HPO_4_/NaH_2_PO_4_ aqueous solution.

In order to further understand why the Au layer, not the Pd layer, can inhibit the faradaic reaction of MoS_2_, the electrochemical behavior of Pd and Au were measured and the results are shown in Fig. 4a. According to previous studies^34,35^, the faradaic potential window is defined as a potential range in which a material exhibits stable and reversible electrochemical faradaic redox reactions in the electrolyte. Therefore, a Pd electrode indicates a reduction faradaic potential window^16,36,37^, which is assigned as Pd + x H^+^ + x e^−^ ↔ β-PdH_x_ (1). Different from a Pd electrode, an Au electrode only exhibits an oxidation faradaic potential window, Au + H_2_O ↔ Au-O + 2 H^+^ + 2 e^−^ (2). Since MoS_2_ is reduced by photo-generated electrons in CCZTS, enlarged reduction faradaic potential windows of Pd and Au are shown in Fig. 4b. A Pd electrode indicates a reduction faradaic potential window of 0 V_RHE_ to 0.45 V_RHE_, while the Au electrode indicates a negligible reduction faradaic capacitance at the same potential window. It is because the lower adsorption energy of H^+^ on the surface of Pd than on the Au layer, which leads to a much higher capacitance of Pd than Au^38,39^. In order to investigate the effects of the coating of Pd and Au on the faradaic capacitance of MoS_2_ (Supplementary Fig. 24), the cyclic voltammetry curves of MoS_2_, MoS_2_/Pd and MoS_2_/Au were measured in the same electrolyte and the results are shown in Fig. 4c. The faradaic capacitance of MoS_2_ increases after the coating of a Pd layer, but decreases obviously after coating the Au layer. In order to further elucidate the reasons of Au not Pd inhibiting Na^+^ intercalation, in situ electrochemical Raman spectroscopy was carried out to investigate the change of MoS_2_, MoS_2_/Pd and MoS_2_/Au samples during electrochemical measurement and the experimental details are shown in Supplementary Fig. 25. The Raman peak intensity of MoS_2_ does not change obviously at the potential of 0.5 V_RHE_, and decreases from 0.4 V_RHE_ to negligible at −0.2 V_RHE_ (Fig. 4d). Therefore, Na^+^ intercalation in MoS_2_ becomes more intense with the potential shifting negatively, consistent with the previous study^33^. The Raman peak intensity of MoS_2_ in a MoS_2_/Pd sample also decreases with the potential shifting negatively, but changes only slightly in MoS_2_/Au (Fig. 4e, f). In a MoS_2_/Pd sample, after the reduction adsorption of H^+^ on Pd (Pd + x H^+^ + x e^−^ ↔ β-PdH_x_), interface charges can transfer from Pd to MoS_2_ following the reaction of MoS_2_ + x Na^+^ + β-PdH_x_ ↔ Na_x_MoS_2_ + Pd + x H^+^. In contrast, there is negligible reduction adsorption of H^+^ on an Au layer, which leads to Na^+^ intercalation being suppressed into MoS_2_. Therefore, the Au layer plays the role of an ion blocking layer in the heterojunction photocathode. In order to further confirm the effects of an ion blocking layer, the photoelectrocatalytic performances of the MoS_2_/CCZTS, MoS_2_/Pd/CCZTS and MoS_2_/Au/CCZTS photocathodes were also measured in the electrolytes with different alkali metal ions (Na^+^/K^+^/Cs^+^) (Supplementary Figs. 26–29). The results suggest that the intercalation reaction of MoS_2_ can also be suppressed with increasing alkali metal ion radii in the electrolytes and improve the photoelectrocatalytic performance of the three heterojunction photocathodes. Especially, the MoS_2_/Au/CCZTS photocathode indicates a high photocurrent of 16.3 mA/cm^2^ at 0 V_RHE_ in the electrolyte with Cs^+^, which is close to the highest value in the neutral aqueous solution (Supplementary Table 4). Therefore, the photoelectrocatalytic performance of the heterojunction photocathodes can be improved remarkably by regulating ion transfer. So far, there are some reports on improving the performance of photoelectrocatalytic cells and dye-sensitized solar cells by adjusting the concentrations and species of ions in the electrolytes^40,41^. However, most of these previous studies only focus on the effects of various electrolytes on the properties of an individual catalyst or semiconductor, but not interface charge transfer in the heterojunctions. This work provides a different paradigm of regulating ion transfer to design high-performance semiconductor heterojunctions.

### Potential window alignment to improve photoelectrocatalytic performance in a MoS_2_/Au/CCZTS heterojunction photocathode

**Fig. 5:**
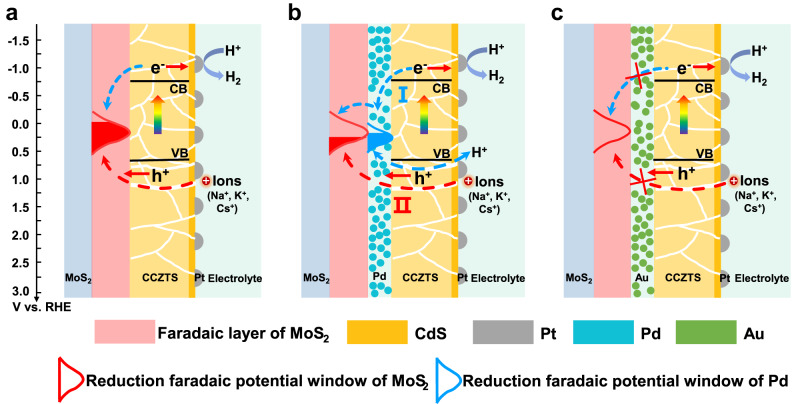
The mechanism of Au to improve the photoelectrocatalytic performance of a MoS_2_/CCZTS heterojunction photocathode. **a**–**c** Interface charge transfer in MoS_2_/CCZTS (**a**), MoS_2_/Pd/CCZTS (**b**) and MoS_2_/Au/CCZTS (**c**) heterojunction photocathodes after coating a CdS buffer layer and a Pt co-catalyst.

According to the above results and analysis, we propose a mechanism for the improved performance of MoS_2_/CCZTS after introducing the Au intermediate layer. According to the above results (Supplementary Figs. 9, 10, 24), the band positions of semiconductors and the faradaic potential windows of MoS_2_, Pd and Au are plotted in Fig. 5. When the conduction band of CCZTS is higher than the faradaic potential window of MoS_2_, part of the photo-generated electrons in CCZTS can reduce H^+^ into hydrogen, and the other electrons reduce the MoS_2_ with the intercalation of Na^+^ (K^+^/Cs^+^) from the electrolyte. Moreover, part of photo-excited holes can re-oxidize the Na_x_MoS_2_ (K_x_MoS_2_ or Cs_x_MoS_2_) during the transfer process. Therefore, the MoS_2_ plays the role of recombination centers, which decreases the photocurrent of the MoS_2_/CCZTS sample (Fig. 5a). Since a Pd intermediate layer indicates a similar reduction faradaic window position with MoS_2_, the photo-generated electrons in CCZTS can transfer to Pd and induce the H^+^ adsorption reaction to form β-PdH_x_, which can further reduce MoS_2_ and is recovered into Pd. The charge transfer at MoS_2_/Pd interface follows the reaction of MoS_2_ + x Na^+^ (K^+^/Cs^+^) + β-PdH_x_ ↔ Na_x_MoS_2_ (K_x_MoS_2_/Cs_x_MoS_2_) + Pd + x H^+^, which leads to a higher capacitance and a lower photocurrent in MoS_2_/Pd/CCZTS. However, the Au intermediate layer can inhibit the Na^+^ intercalation into MoS_2_ and improve the performance of the MoS_2_/CCZTS sample. These results suggest that a faradaic potential window, not a Fermi level, is a criterion for interface charge transfer direction in a faradaic junction. The driving force for charge separation in a faradaic junction is the difference of electrochemical potential at the interface, which is intrinsically different from the built-in electric field in a physical heterojunction based on classic band theory^9–16^.

## Discussion

In summary, by introducing the Pd and Au intermediate layers in MoS_2_/CCZTS, the photocurrent increases obviously in MoS_2_/Au/CCZTS, but decreases slightly in MoS_2_/Pd/CCZTS. It is because the Au layer acts as an ion blocking layer to inhibit the recombination of photo-generated carriers and improve the photocurrent. Moreover, we find that it is the potential windows not the band positions of the intermediate layer that is the criterion for controlling interface charge transfer direction in a faradaic junction. The potential window alignment theory cannot only deepen the understanding of interface charge transfer in the heterojunction but also offer a suitable design concept for improving the performance of other solar energy conversion and storage devices, such as photocatalysis, photoelectrocatalysis, photoelectrostorage and third-generation photovoltaics.

## Methods

### Preparation of the precursor solution

The precursor solution was prepared in a glovebox with O_2_ and H_2_O level below 5 ppm at room temperature similar to previous reports^19,42^. First, 4 mL of DMSO (99.8%, J&K) was added to a glass vial by a syringe followed by adding 2.4785 g of thiourea (Tu, 99%, Aladdin, recrystallized twice) and 0.7405 g of CuCl (99.999%, Alfa); Second, 1.0420 g of SnCl_4_ (99.99%, Alfa) was added to a second vial followed by adding 4 mL of DMSO, 0.6165 g of Zn(OAc)_2_ (99.99%, Aladdin), and 0.2638 g of CdCl_2_ (99.99%, Aladdin). The two vials were mixed and stirred until clear. The mole ratios of the elements in the solution are Cu/(Zn + Cd+Sn) = 0.85, (Zn + Cd)/Sn = 1.20, Cd/(Zn + Cd) = 0.30.

### Fabrication of the CCZTS absorber film

The glass/Mo/Pd and glass/Mo/Au substrates were prepared by depositing Pd films or Au films on Mo glass substrates using an ion beam sputtering method at room temperature for 15 min. To obtain CCZTS precursor thin film, the precursor solution was spin coated on glass/Mo, glass/Mo/Pd, or glass/Mo/Au substrates at 2000 rpm for 60 s. After spin-coating, the films were immediately annealed at 370 °C for 90 s on a hot plate and then cooled down. The coating and annealing were repeated seven times in order to obtain a desired absorber layer thickness in the glovebox. The films were placed in a graphite box with 100 mg of sulfur powder and 100 mg of stannous sulfide and the graphite box was placed in a rapid thermal processing (RTP) tube furnace for sulfurization. The tube was pumped and refilled with Argon three times before heated up. The furnace was heated to 620 °C from room temperature over a period of 20 min. The sulfurization was performed at this temperature for 15 minutes and cooled to room temperature naturally.

### Coating of a CdS buffer layer and a Pt co-catalyst on CCZTS films

A CdS buffer layer was deposited on the CCZTS surface by a chemical bath deposition method. Specifically, the CCZTS samples were immersed in aqueous solution containing 1.5 mM CdSO_4_, 75 mM SC(NH_2_)_2_ and 2.3 M NH_3_·H_2_O. The deposition temperature was 60 °C and the deposition time was 5 min. Then, a Pt co-catalyst was deposited on the surface of CdS/CCZTS using an ion beam sputtering method at room temperature for 25 s.

### Characterization of samples

The crystalline structures of the three samples were characterized by X-ray diffraction (XRD smartlab, 9 kW). After CCZTS were scraped off the substrate with a tweezer, the MoS_2_, MoS_2_/Pd and MoS_2_/Au substrates were characterized by Grazing Incidence X-Ray Diffraction (GIXRD) (Rigaku Smart lab X-ray diffractometer), four-point probe (Summit 11000 m) and scanning electron microscope (SEM, Gemini500). The cross-section and top-view morphologies of the MoS_2_/CCZTS, MoS_2_/Pd/CCZTS and MoS_2_/Au/CCZTS samples were observed by SEM. The element distribution of the thick and dense CCZTS samples before and after illumination in the electrolyte were identified by time-of-flight secondary ion mass spectrometry (TOF-SIMS 5 iontof, PHI NanoTOFII). The absorption spectra of the samples were recorded by a UV-visible-NIR spectrophotometer (PE lambda 950). The Mott-Schottky plots of CCZTS and MoS_2_ samples were measured in a cell with a carbon rod and a saturated Ag/AgCl electrode as the counter and reference electrode, respectively. An electrochemical analyzer (CHI 760e, Shanghai Chenhua) was used to control the potentials of CCZTS and MoS_2_ samples with an a.c. amplitude of 5 mV and a frequency of 1000 Hz. For ultraviolet photoelectron spectra (UPS) measurement, the Pd and Au films were deposited on glass substrates by an ion beam sputtering method for 15 min and the photoelectron spectra of the films were performed in a Kratos Ultra Spectrometer (K-ALPHA+, Thermo Fisher) by using a He I (21.22 eV) discharge lamp.

### In situ irradiated XPS, in situ electrochemical Raman and quasi in situ irradiated Raman characterization of samples

In situ irradiated X-ray photoelectron spectra were measured under a full arc Xe lamp with a XPS spectrometer (Thermofisher Escalab 250Xi). Since the CCZTS layer can attenuate XPS intensity of MoS_2_, a thin and porous CCZTS were prepared by coating and annealing for two times. For in situ irradiated XPS measurement, the MoS_2_/CCZTS, MoS_2_/Pd/CCZTS and MoS_2_/Au/CCZTS samples were pretreated by immersing them in 0.2 M Na_2_HPO_4_/NaH_2_PO_4_ aqueous solution and exposing them to water vapor. In order to investigate the charge transfer in the three samples, in situ XPS was measured in the dark and after 10-mins’ illumination. In situ electrochemical Raman spectra (Horiba T64000, excitation wavelength ~488 nm) were measured in a cell with a saturated Ag/AgCl electrode and a Pt wire as a reference and a counter electrode, respectively. The potential of the MoS_2_, MoS_2_/Pd and MoS_2_/Au was applied by an electrochemical analyzer (CHI-760E, Shanghai Chenhua). Raman signals were detected simultaneously when the electrochemical curves of the samples were measured in the dark. An aqueous solution of 0.2 M Na_2_HPO_4_/NaH_2_PO_4_ (K_2_HPO_4_/KH_2_PO_4_ or Cs_2_HPO_4_/CsH_2_PO_4_) was used as electrolyte. Quasi in situ irradiated Raman spectra of thick and dense CCZTS samples on different substrates were measured before and after illumination by a Xe lamp in 0.2 M Na_2_HPO_4_/NaH_2_PO_4_ aqueous solution.

### Photoelectrochemical measurement

The photoelectrochemical performance was measured in a conventional three-electrode cell using an electrochemical analyzer (CHI-760E, Shanghai Chenhua) with an AM 1.5 G sunlight simulator (CEL-AAAS50, 100 mW/cm^2^, China Education Au-light). A carbon rod and a saturated Ag/AgCl electrode were employed as the counter and the reference electrode, respectively. The electrolyte was the aqueous solution of 0.2 M Na_2_HPO_4_/NaH_2_PO_4_ (K_2_HPO_4_/KH_2_PO_4_ and Cs_2_HPO_4_/CsH_2_PO_4_). The potentials of working electrode were converted to reversible hydrogen electrode by the following formula: V_RHE_ = V_Ag/AgCl_ + 0.199 V + 0.059 * pH. The incident photon-to-current efficiency (IPCE) was obtained under irradiation of different wavelengths of light and was calculated by the following formula:

$${IPCE}=\frac{1240*{I}_{{ph}}}{P*\lambda }$$

Where the I_ph_ is the photocurrent density obtained at the potential of 0 V_RHE_, P and λ are the power density (μW/cm^2^) and wavelength (μW/cm^2^) of the incident light, respectively.

### Supplementary information

Supplementary Information

Peer Review File

## Data Availability

All data are available in the main text or the Supplementary Information files. Additional data related to the findings of this study are available from the corresponding author upon reasonable request.
