# Supplementary material for: Potential window alignment regulating ion transfer in faradaic junctions for efficient photoelectrocatalysis
Source: Nat Commun. 2023 Dec 2;14:7969. doi: 10.1038/s41467-023-43916-6 (PMC10693569; doi:10.1038/s41467-023-43916-6)
Supplement: Supplementary file 1 — Supplementary Information [file 41467_2023_43916_MOESM1_ESM.pdf]

Supplementary Information

Potential window alignment regulating ion transfer in faradaic junctions for efficient photoelectrocatalysis

Hongzheng Dong[1]†, Xiangyu Pan[2]†, Yuancai Gong[2], Mengfan Xue[3], Pin Wang[3], SocMan Ho-Kimura[4], Yingfang Yao[1], Hao Xin[2,*], Wenjun Luo[1,*], Zhigang Zou[1,3]

[1]Eco-materials and Renewable Energy Research Center (ERERC), National Laboratory of Solid State Microstructures, College of Engineering and Applied Sciences, Nanjing University, Nanjing 210093, China.

[2]State Key Laboratory of Organic Electronics and Information Displays & Institute of Advanced Materials, Nanjing University of Posts & Telecommunications, Nanjing 210023, China.

[3]Eco-materials and Renewable Energy Research Center (ERERC), Jiangsu Key Laboratory for Nano Technology, National Laboratory of Solid State Microstructures and Department of Physics, Nanjing University, Nanjing 210093, China.

[4]Institute of Applied Physics and Materials Engineering, University of Macau, Macau SAR, China.

†These authors contributed equally to this work.

[*]Corresponding authors. Email: wjluo@nju.edu.cn; iamhxin@njupt.edu.cn

This PDF file includes:

Supplementary Figure 1 to Figure 29
Supplementary Table 1 to Table 4
Supplementary References

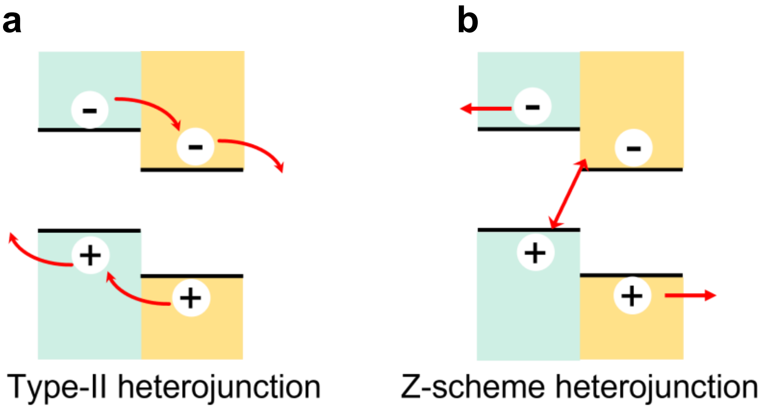

Supplementary Fig. 1 Interface charge transfer in various types of semiconductor junctions. The diagram of interface charge transfer in Type-II (**a**) and Z-scheme (**b**) heterojunction.

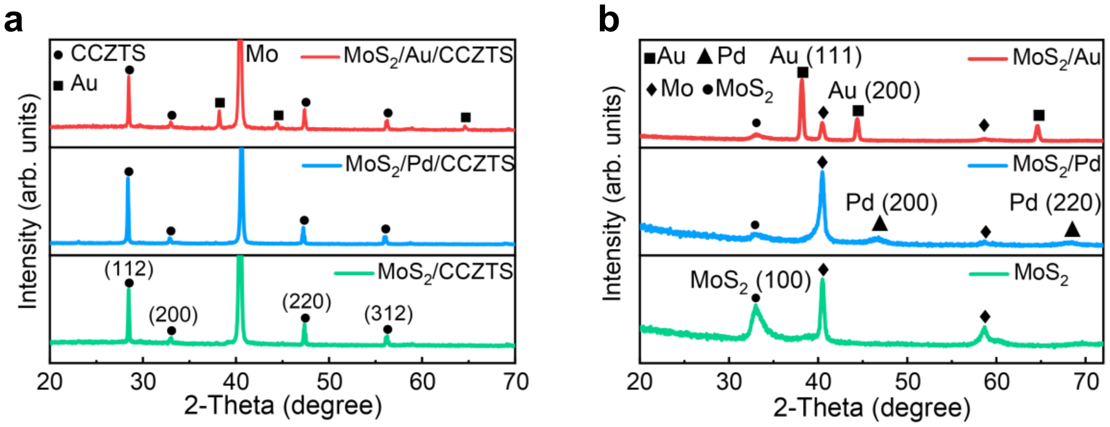

Supplementary Fig. 2 XRD characterization. The XRD patterns (**a**) of MoS_2/CCZTS, MoS_2/Pd/CCZTS and MoS_2/Au/CCZTS. The GIXRD patterns (**b**) of MoS_2, MoS_2/Pd and MoS_2/Au substrates.

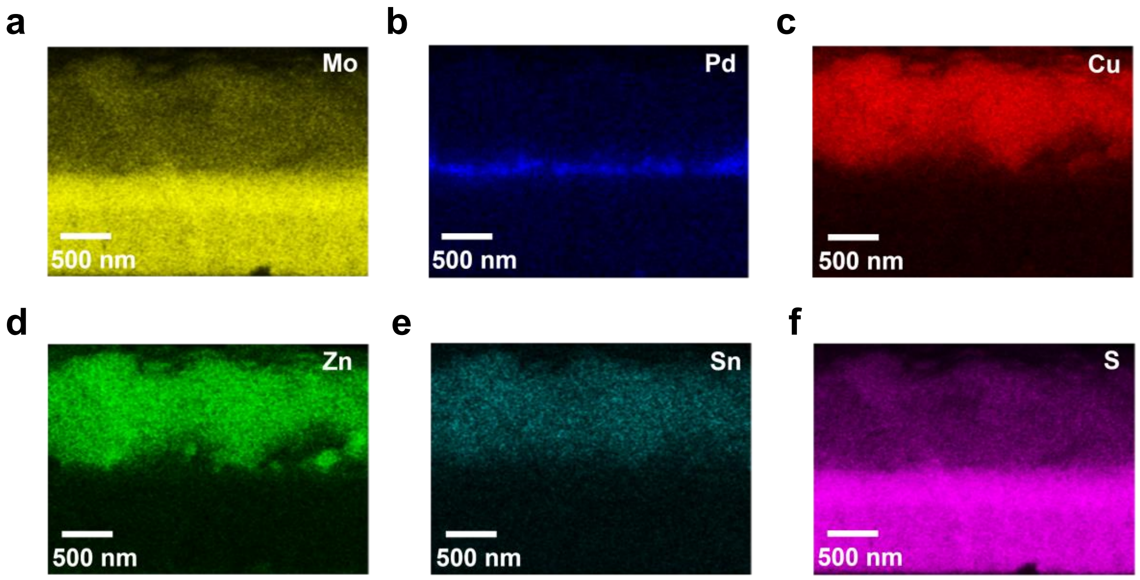

Supplementary Fig. 3 EDS characterization of MoS_2/Pd/CCZTS. Element mapping of Mo (**a**), Pd (**b**), Cu (**c**), Zn (**d**), Sn (**e**), and S (**f**), respectively.

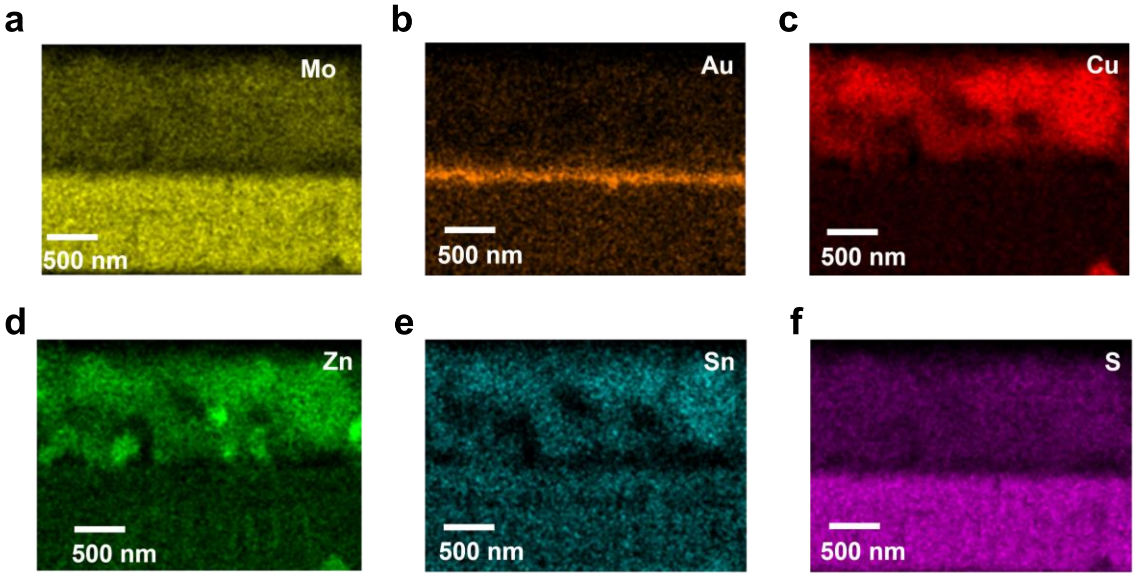

Supplementary Fig. 4 EDS characterization of MoS_2/Au/CCZTS. Element mapping of Mo (**a**), Au (**b**), Cu (**c**), Zn (**d**), Sn (**e**), and S (**f**), respectively.

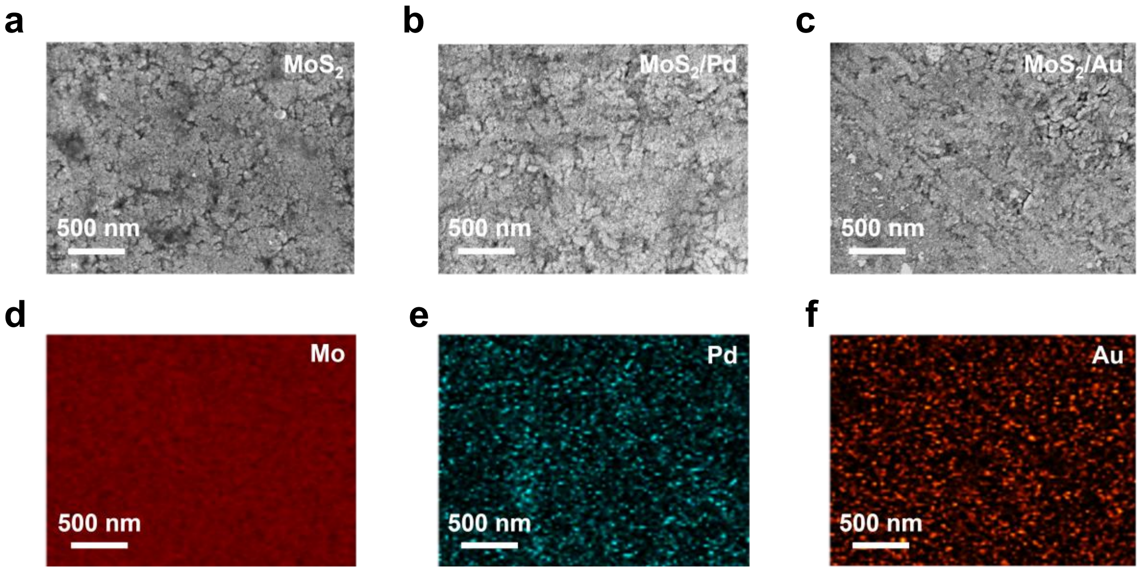

Supplementary Fig. 5 SEM and EDS characterization. Top-view SEM images (**a-c**) and corresponding EDS characterization (**d-f**) of MoS_2, MoS_2/Pd and MoS_2/Au.

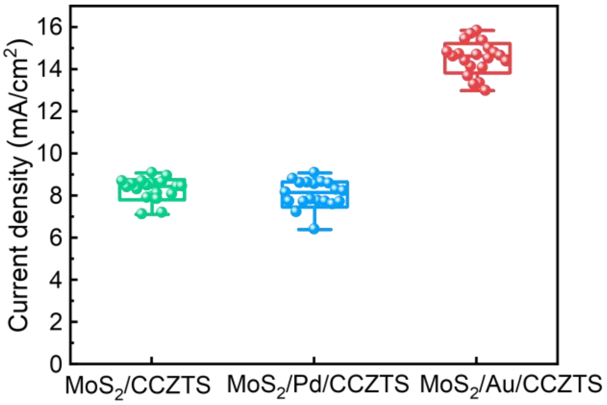

Supplementary Fig. 6 Statistical box data of photocurrent density @ 0 V_{RHE} of MoS_2/CCZTS, MoS_2/Pd/CCZTS and MoS_2/Au/CCZTS photoelectrodes after coating a CdS buffer layer and a Pt co-catalyst. The box plot denotes median (center line), 25th (bottom edge of the box), 75th (top edge of the box), 95th (upper whisker) and 5th (lower whisker) percentiles. The sample size in each column is 20 photoelectrodes. Electrolyte: 0.2 M Na_2HPO_4/NaH_2PO_4 aqueous solution, light source: an AM 1.5G sunlight simulator, light intensity: 100 mW/cm^2.

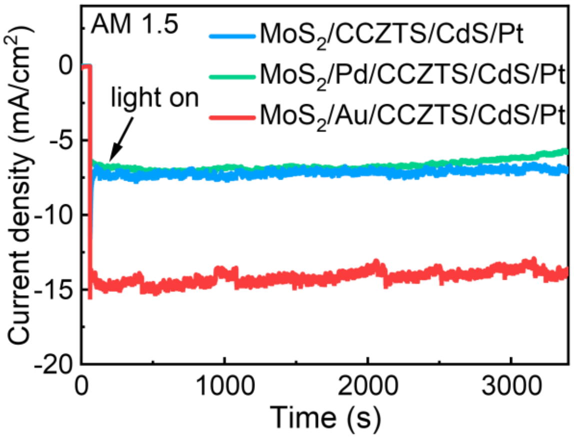

Supplementary Fig. 7 Photocurrent density-time curves @ 0 V_{RHE} of MoS_2/CCZTS, MoS_2/Pd/CCZTS and MoS_2/Au/CCZTS photoelectrodes after coating a CdS buffer layer and a Pt co-catalyst. Electrolyte: 0.2 M Na_2HPO_4/NaH_2PO_4 aqueous solution, light source: an AM 1.5G sunlight simulator, light intensity: 100 mW/cm^2.

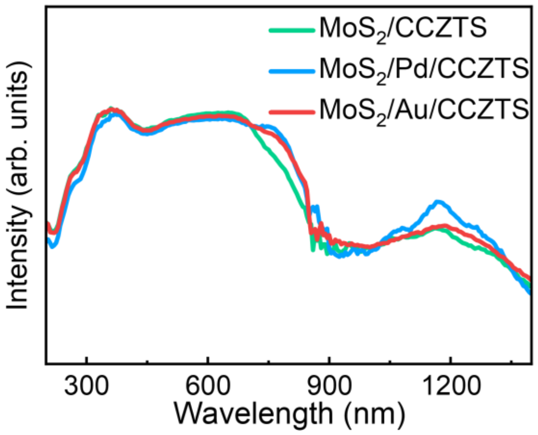

Supplementary Fig. 8 Absorption spectra of MoS_2/CCZTS, MoS_2/Pd/CCZTS and MoS_2/Au/CCZTS photoelectrodes.

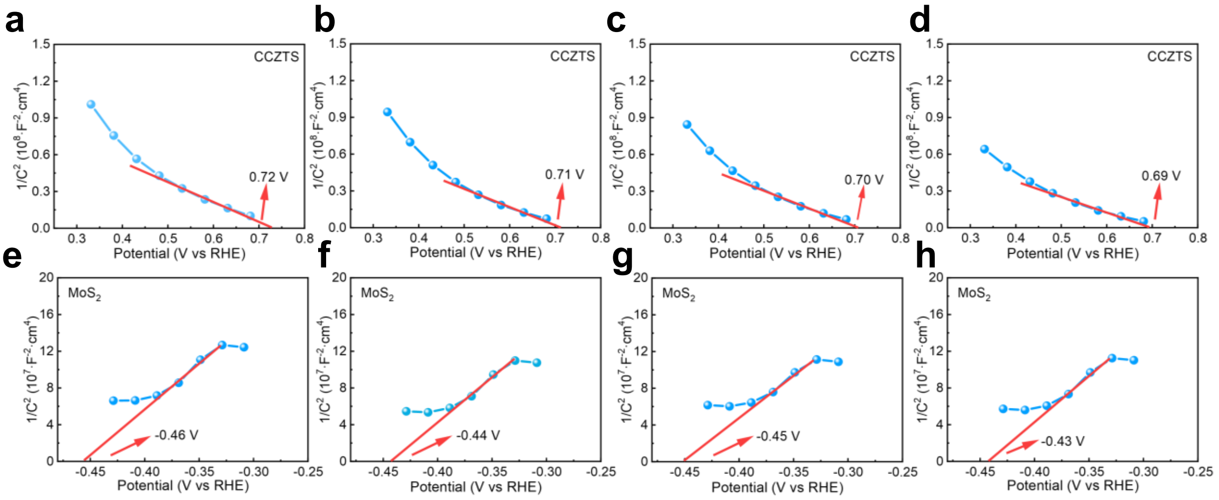

Supplementary Fig. 9 Mott-Schottky characterization. Mott-Schottky plots of four CCZTS samples (**a-d**) and four MoS_2 samples (**e-h**) in 0.2 M Na_2HPO_4/NaH_2PO_4 aqueous solution in the dark.

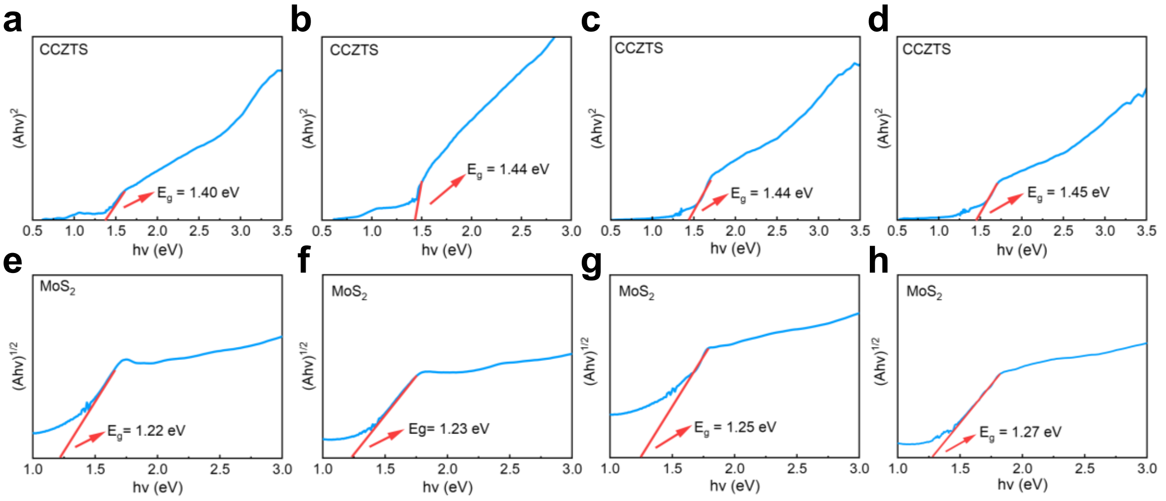

Supplementary Fig. 10 UV-visible absorption spectra characterization. Tauc plots of UV-visible absorption spectra of four CCZTS samples (**a-d**) and four MoS_2 samples (**e-h**); A, absorption coefficient; h, Planck's constant; v, photon's frequency.

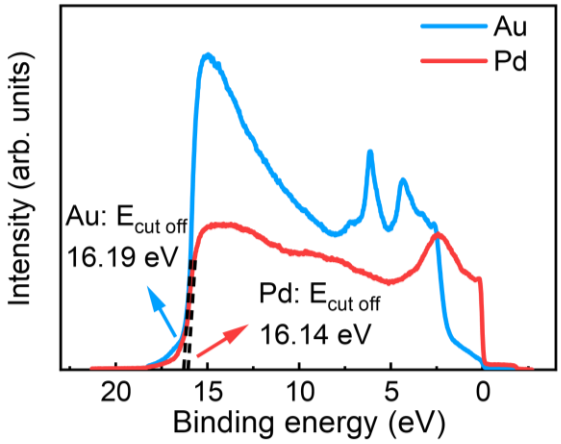

Supplementary Fig. 11 Ultraviolet photoelectron spectra of Au and Pd films on glass substrates after Ar^+ sputter-cleaning (3000 V for 15 minutes).

The work functions of Pd and Au are calculated to be 5.08 eV and 5.03 eV by subtracting the energy difference between the analyser Fermi levels and the binding energies of the secondary electron cutoffs ($E_{cut\ off}$) from the excitation energy of He I excitation energy (21.22 eV)[1]. The results are shown in Supplementary Table 3. The work functions of Pd and Au particulate films in this study are similar to the reference values in previous studies[2,3]. The Fermi levels in the reversible hydrogen electrode (RHE) scale can be calculated from the work functions in the absolute vacuum scale by the formula of $eV_{RHE} = E_{Vacuum} - 4.5$ eV[4], also summarized in Supplementary Table 3.

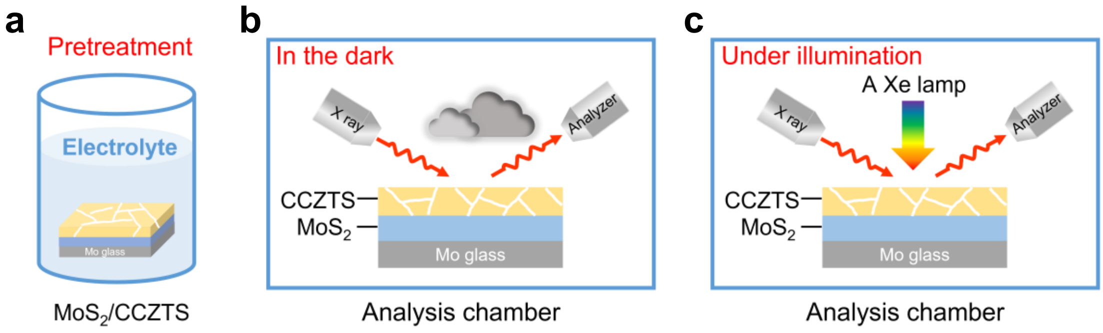

Supplementary Fig. 12 Schematic illustration of the experimental details for in situ irradiated XPS. The samples were immersed in 0.2 M Na_2HPO_4/NaH_2PO_4 aqueous solution for pretreatment (**a**). The XPS signals of the samples were collected in the dark (**b**) and under full arc Xe lamp illumination (**c**).

For in situ XPS measurement, the heterojunction samples were pretreated by immersing them in 0.2 M Na_2HPO_4/NaH_2PO_4 aqueous solution (Supplementary Fig. 12a) and were then transferred to the chamber for XPS measurement in the dark (Supplementary Fig. 12b) and under illumination (Supplementary Fig. 12c).

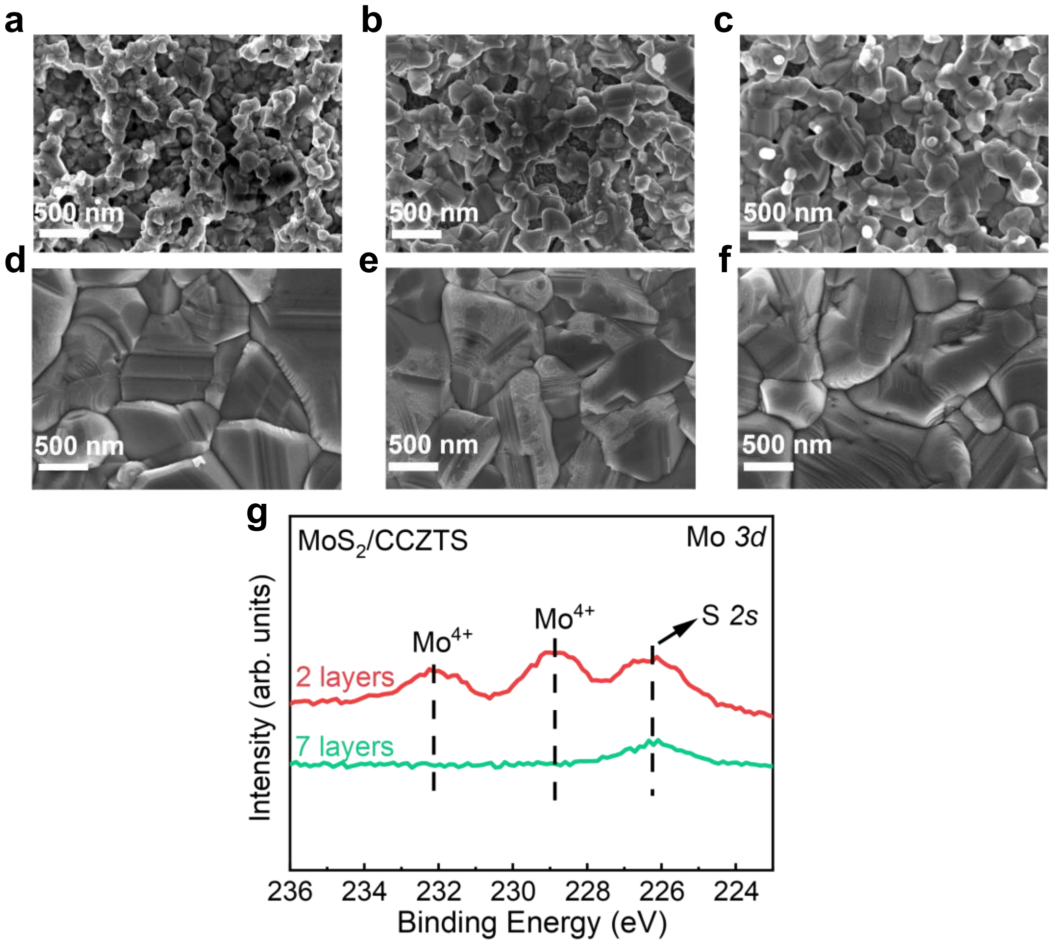

Supplementary Fig. 13 SEM and XPS characterization. Top-view SEM images of CCZTS (two layers) on MoS_2 (**a**), MoS_2/Pd (**b**) and MoS_2/Au (**c**); CCZTS (seven layers) on MoS_2 (**d**), MoS_2/Pd (**e**) and MoS_2/Au (**f**). The XPS spectra (**g**) of Mo *3d* in the MoS_2/CCZTS (two layers) and MoS_2/CCZTS (seven layers) heterojunctions.

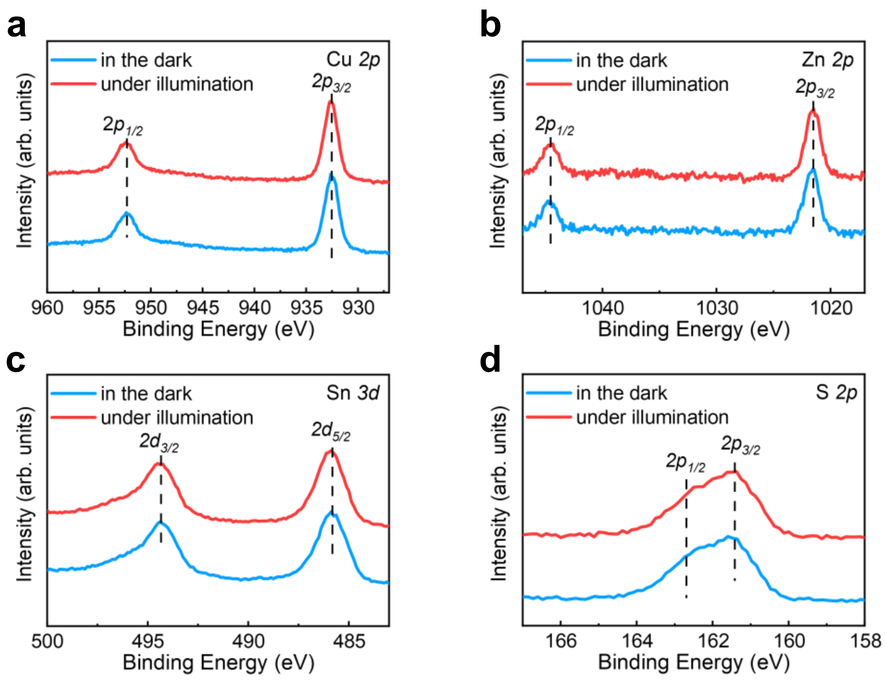

Supplementary Fig. 14 In situ irradiated XPS spectra characterization. In situ irradiated XPS spectra of Cu *2p* (**a**), Zn *2p* (**b**), Sn *3d* (**c**) and S *2p* (**d**) in the MoS_2/CCZTS heterojunction in the dark and under Xe lamp illumination.

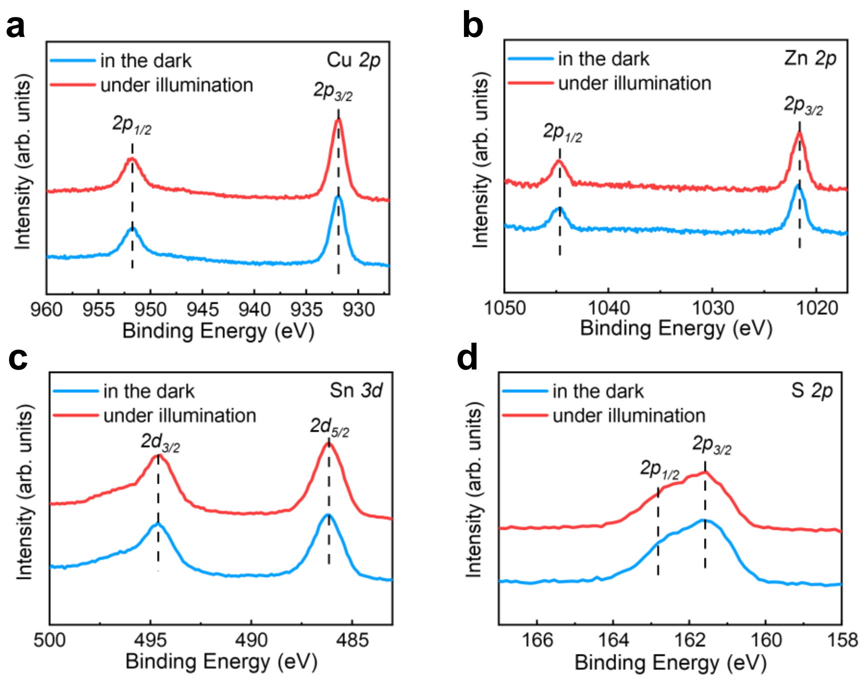

Supplementary Fig. 15 In situ irradiated XPS spectra characterization. In situ irradiated XPS spectra of Cu *2p* (**a**), Zn *2p* (**b**), Sn *3d* (**c**) and S *2p* (**d**) in the MoS_2/Pd/CCZTS heterojunction in the dark and under Xe lamp illumination.

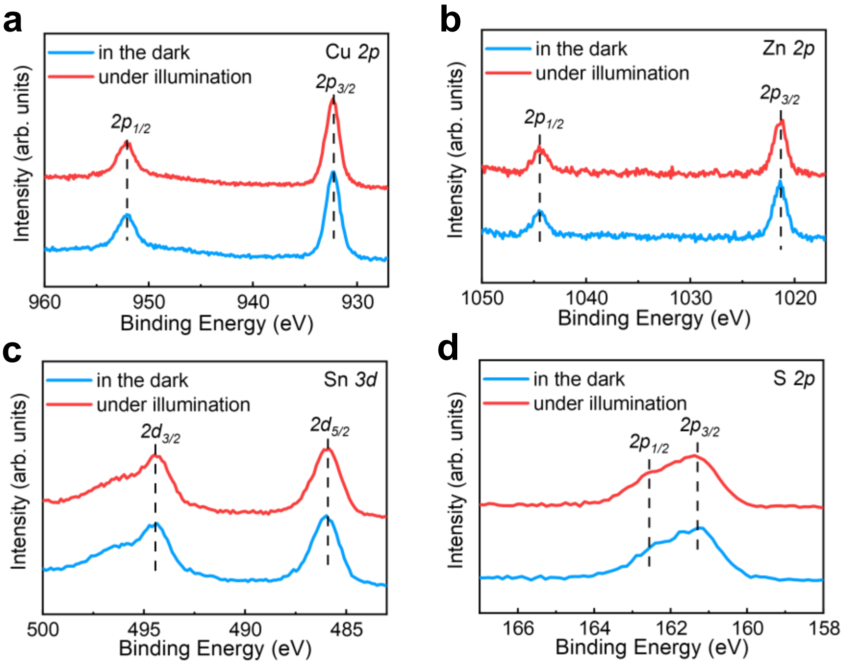

Supplementary Fig. 16 In situ irradiated XPS spectra characterization. In situ irradiated XPS spectra of Cu *2p* (**a**), Zn *2p* (**b**), Sn *3d* (**c**) and S *2p* (**d**) in the MoS_2/Au/CCZTS heterojunction in the dark and under Xe lamp illumination.

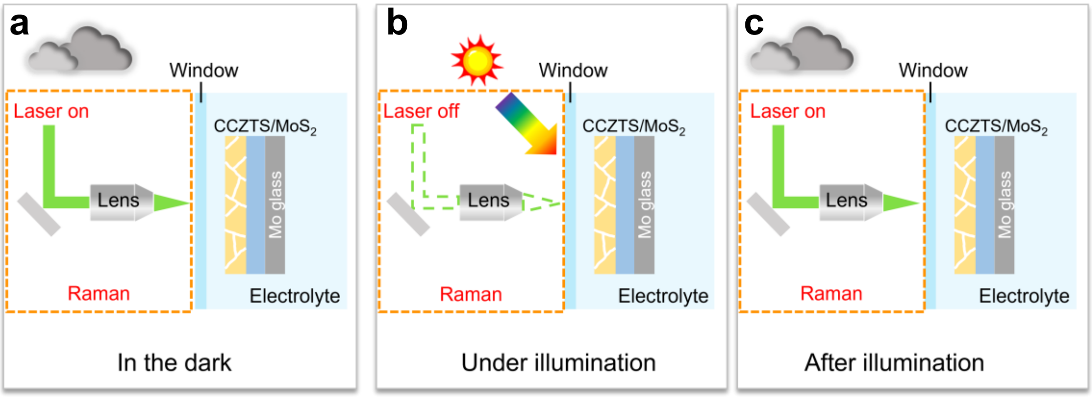

Supplementary Fig. 17 Schematic illustration of the experimental setup for quasi in situ irradiated Raman in the electrolytes under illumination. The Raman spectra of the samples were collected in 0.2 M Na_2HPO_4/NaH_2PO_4 aqueous solution in the dark (**a**), and then the samples were exposed to a Xe lamp for 10 minutes (**b**), the Raman characterization was carried out on the samples immediately after illumination (**c**).

Since the light source has an effect on the Raman signals, in situ irradiated Raman cannot be used to investigate the interface charge transfer in the heterojunctions under illumination. Therefore, quasi in situ irradiated Raman was used and the measurement process is detailed as follows. The heterojunction samples were measured firstly in 0.2 M Na_2HPO_4/NaH_2PO_4 aqueous solution in the dark by Raman (Supplementary Fig. 17a) and then were illuminated under a Xe lamp for 10 minutes (Supplementary Fig. 17b), during which the laser in Raman was off. After illumination, Raman characterization was carried out on the samples immediately (Supplementary Fig. 17c).

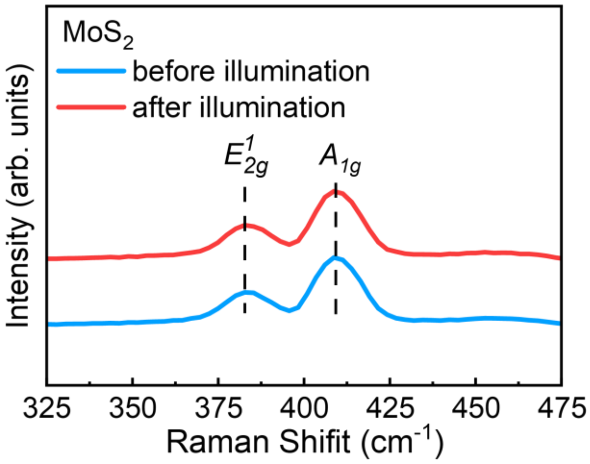

Supplementary Fig. 18 Quasi in situ irradiated Raman spectra of MoS_2 in 0.2 M Na_2HPO_4/NaH_2PO_4 aqueous solution (pH=6.5) before and after illumination under Xe lamp.

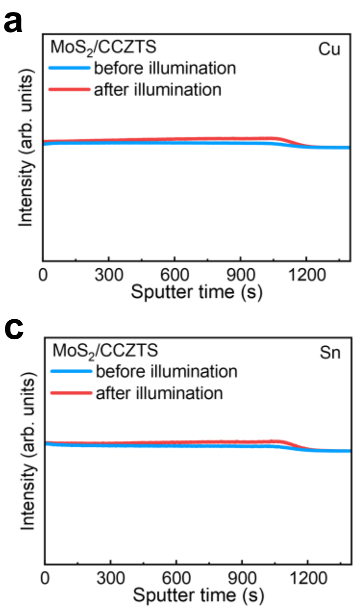

Supplementary Fig. 19 Time-of-flight secondary-ion mass spectrometry characterization. Secondary ion intensities of Cu (**a**), Zn (**b**), Sn (**c**) and S (**d**) ions in the MoS_2/CCZTS heterojunction before and after Xe lamp illumination. Electrolyte: 0.2 M Na_2HPO_4/NaH_2PO_4 aqueous solution; Light source: a full arc Xe lamp.

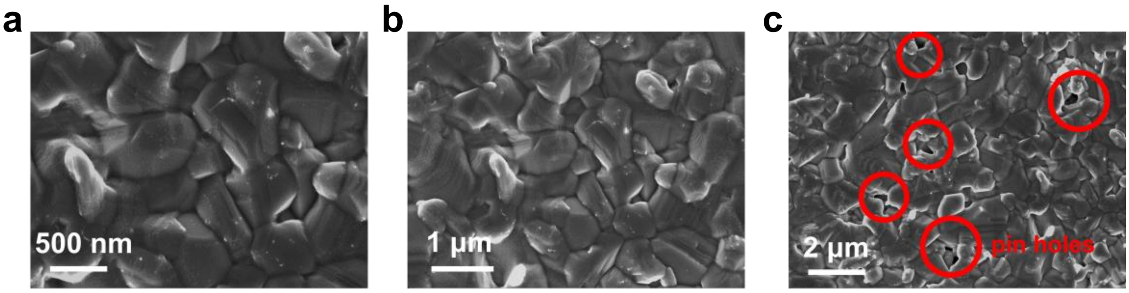

Supplementary Fig. 20 SEM characterization. Top-view SEM images of CCZTS on MoS_2 at different scales of 500 nm (**a**), 1 μm (**b**) and 2 μm (**c**).

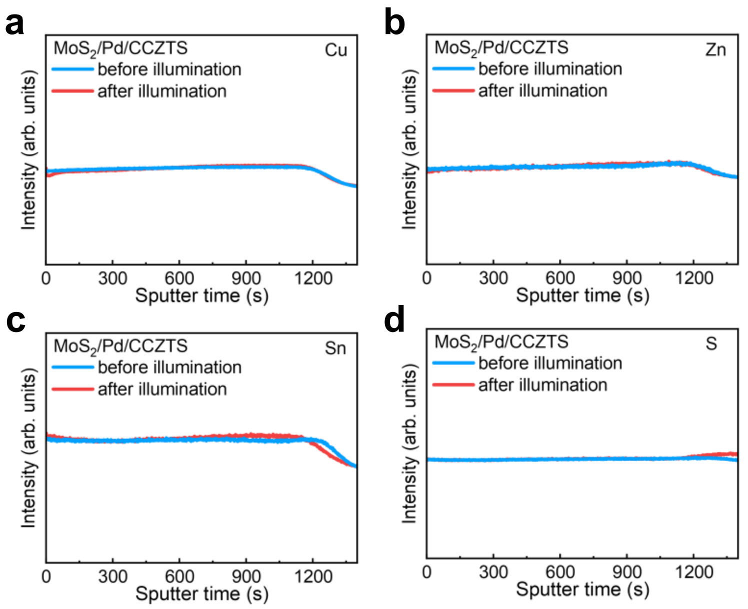

Supplementary Fig. 21 Time-of-flight secondary-ion mass spectrometry characterization. Secondary ion intensities of Cu (**a**), Zn (**b**), Sn (**c**) and S (**d**) ions in the MoS_2/Pd/CCZTS heterojunction before and after Xe lamp illumination. Electrolyte: 0.2 M Na_2HPO_4/NaH_2PO_4 aqueous solution; Light source: a full arc Xe lamp.

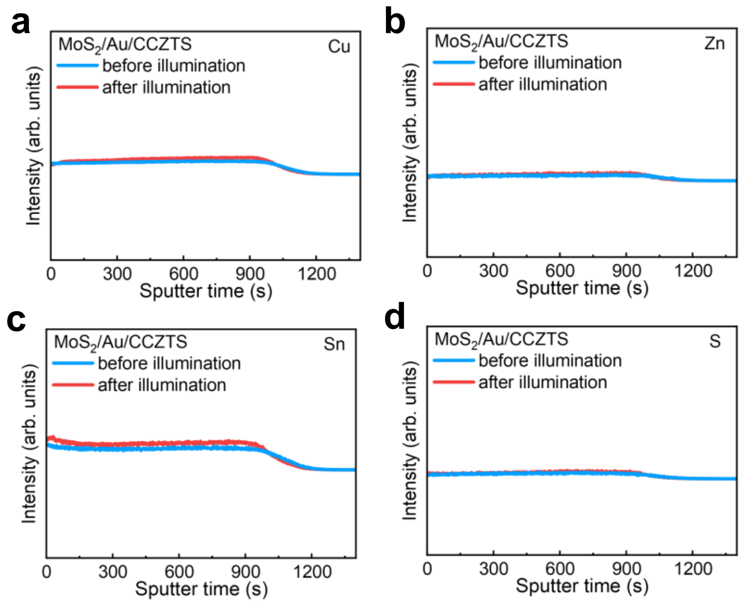

Supplementary Fig. 22 Time-of-flight secondary-ion mass spectrometry characterization. Secondary ion intensities of Cu (**a**), Zn (**b**), Sn (**c**) and S (**d**) ions in the MoS_2/Au/CCZTS heterojunction before and after Xe lamp illumination. Electrolyte: 0.2 M Na_2HPO_4/NaH_2PO_4 aqueous solution; Light source: a full arc Xe lamp.

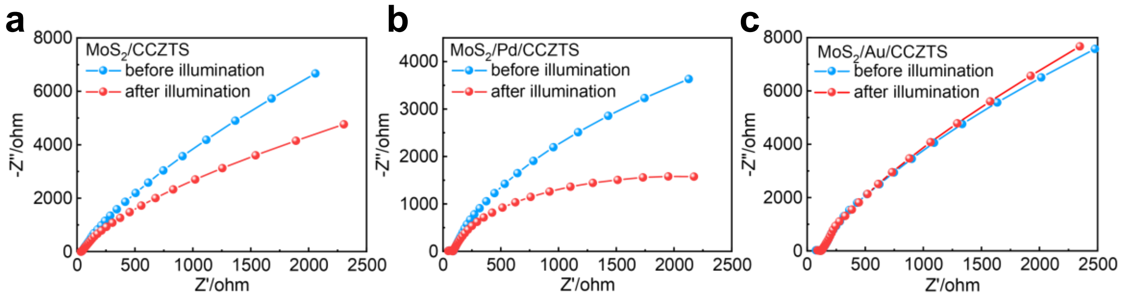

Supplementary Fig. 23 Electrochemical impedance spectroscopy characterization. Electrochemical impedance spectroscopy of MoS_2/CCZTS (**a**), MoS_2/Pd/CCZTS (**b**) and MoS_2/Au/CCZTS (**c**) in 0.2 M Na_2HPO_4/NaH_2PO_4 aqueous solution before and after illumination under Xe lamp, the potential was 0.6 V_{RHE}. Light source: a full arc Xe lamp.

The intercalation reaction will lead to lower resistance in MoS_2. Therefore, electrochemical impedance spectroscopy (EIS) of the three samples was also measured before and after illumination. The circle of MoS_2/CCZTS at low frequency decreases obviously after illumination (Supplementary Fig. 23a). Similar results are obtained in MoS_2/Pd/CCZTS instead of MoS_2/Au/CCZTS sample (Supplementary Figs. 23b-c). The results are in good agreement with XPS, Raman spectra and secondary ion mass spectrometry.

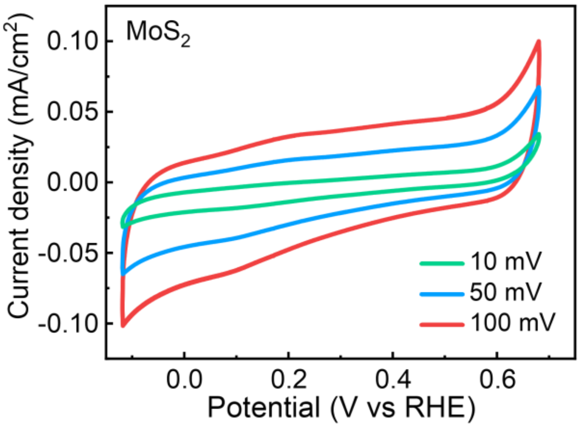

Supplementary Fig. 24 Cyclic voltammetry (CV) curves of MoS_2 in the dark. Electrolyte: 0.2 M Na_2HPO_4/NaH_2PO_4 aqueous solution.

The electrochemical behavior of MoS_2 alone was measured in 0.2 M Na_2HPO_4/NaH_2PO_4 aqueous solution and the results are shown in Supplementary Fig. 24. A series of CV curves with different scan rates exhibit rectangular shape, indicating the capacitance response in the potential window of -0.15 V_{RHE} to 0.65 V_{RHE}[5].

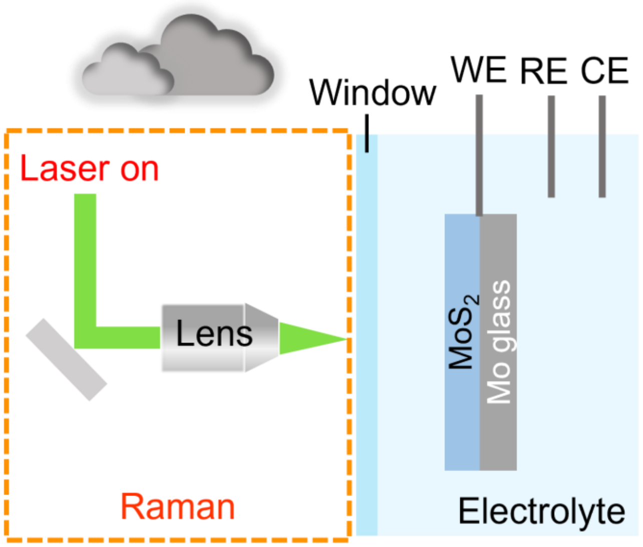

Supplementary Fig. 25 Schematic illustration of the experimental setup for in situ electrochemical Raman during electrochemical measurement in the dark, where WE, RE, and CE represent the working, reference and counter electrodes, respectively.

In situ electrochemical Raman was used to investigate the changes of the samples during electrochemical measurements in the dark. A three-electrode cell was used with the MoS_2, MoS_2/Pd and MoS_2/Au samples as working electrodes (WE), a saturated Ag/AgCl electrode and a Pt wire as a reference (RE) and a counter electrode (CE), respectively (Supplementary Fig. 25). The electrolyte was an aqueous solution of 0.2 M Na_2HPO_4/NaH_2PO_4 (K_2HPO_4/KH_2PO_4 or Cs_2HPO_4/CsH_2PO_4). The potentials of the MoS_2, MoS_2/Pd and MoS_2/Au were controlled by an electrochemical analyzer (CHI-760E, Shanghai Chenhua). Raman signals were detected simultaneously when the electrochemical curves of the samples were measured in the dark.

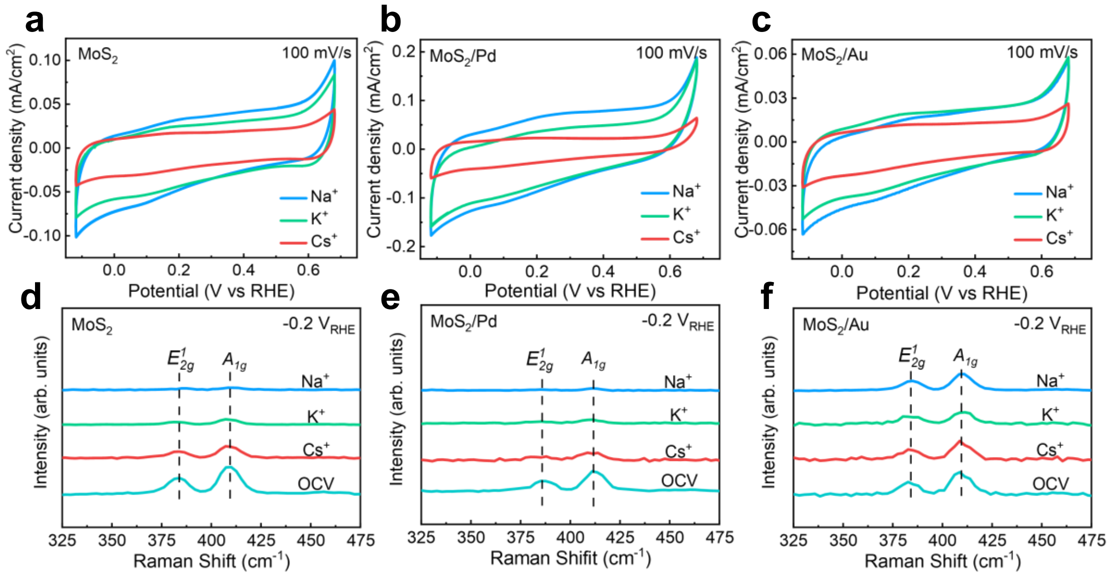

Supplementary Fig. 26 Cyclic voltammetry curves and in situ electrochemical Raman characterization. Cyclic voltammetry curves of MoS_2 (**a**), MoS_2/Pd (**b**) and MoS_2/Au (**c**) in the electrolytes. In situ electrochemical Raman spectra of MoS_2 (**d**), MoS_2/Pd (**e**) and MoS_2/Au (**f**) in the electrolytes at the potential of -0.2 V_{RHE} in the dark. Electrolyte: 0.2 M Na_2HPO_4/NaH_2PO_4, K_2HPO_4/KH_2PO_4 and Cs_2HPO_4/CsH_2PO_4 aqueous solution.

Since Au can inhibit the intercalation reaction in MoS_2 and improve the photoelectrocatalytic performance of MoS_2/CCZTS, alkali metal ions with different radii in the electrolyte can also change the interface charge transfer and photoelectrocatalytic activity of the heterojunction. Supplementary Figs. 26a-c indicate the CV curves of MoS_2, MoS_2/Pd and MoS_2/Au in the different electrolytes. The faradaic capacitance of MoS_2, MoS_2/Pd and MoS_2/Au decreases with increasing alkali metal ion radii in the electrolytes, consistent with previous studies[5]. Moreover, at the potential of -0.2 V_{RHE}, the decrease of Raman peak intensity of MoS_2 in the three samples is much lower in the electrolyte with Cs^+ than that in the electrolytes with Na^+ or K^+ (Supplementary Figs. 26d-f)[6,7]. Therefore, the intercalation in MoS_2 can be inhibited with the increase of alkali metal ion radii in the electrolytes, even under the existence of the Au ion blocking layer.

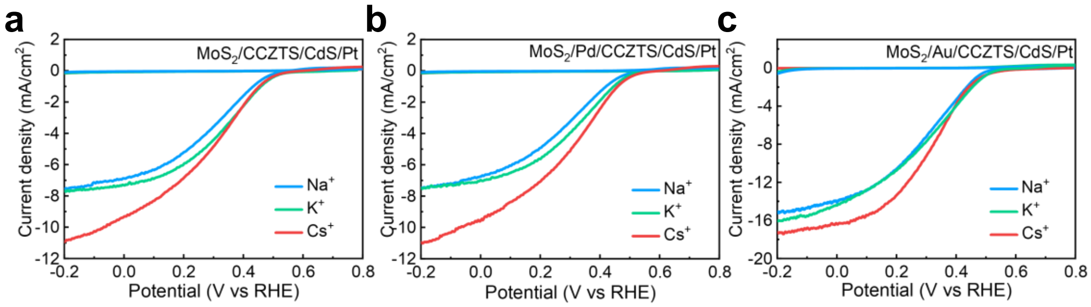

Supplementary Fig. 27 Linear sweep voltammetry test. Linear sweep voltammetry curves of MoS_2/CCZTS (**a**), MoS_2/Pd/CCZTS (**b**) and MoS_2/Au/CCZTS (**c**) after coating a CdS buffer layer and a Pt co-catalyst in the electrolytes. Light source: an AM 1.5G sunlight simulator, light intensity: 100 mW/cm^2. Electrolyte: 0.2 M Na_2HPO_4/NaH_2PO_4, K_2HPO_4/KH_2PO_4 and Cs_2HPO_4/CsH_2PO_4 aqueous solution.

The photocurrent of MoS_2/CCZTS, MoS_2/Pd/CCZTS and MoS_2/Au/CCZTS increases significantly in the electrolyte with Cs^+. Especially, the photocurrent density of MoS_2/Au/CCZTS/CdS/Pt at 0 V_{RHE} increases from 14.9 mA/cm^2 in the electrolyte with Na^+ to 16.3 mA/cm^2 in the electrolyte with Cs^+. In order to confirm the photocurrent is reliable, IPCE action spectra of the MoS_2/Au/CCZTS with CdS and Pt co-catalysts were measured and integrated photocurrent were calculated and the results are shown in Supplementary Fig. 27. The MoS_2/Au/CCZTS photocathode indicates the integrated photocurrent of 16.9 mA/cm^2, which is in good agreement with the experimental value. The obtained photocurrent is close to the highest value among CZTS based photocathodes for solar hydrogen production in neutral aqueous solution (Supplementary Table 4). Therefore, the intercalation reaction of MoS_2 can also be suppressed with increasing alkali metal ion radii in the electrolytes, which can further improve the photoelectrocatalytic performance of the heterojunctions.

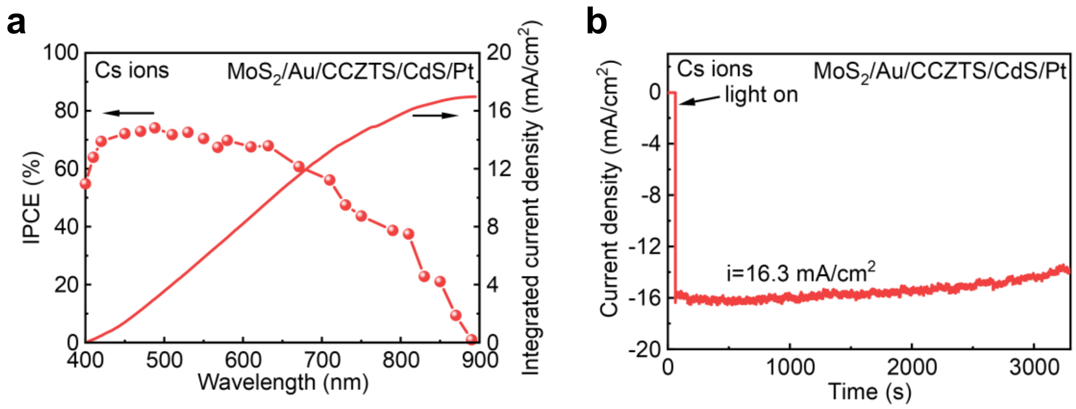

Supplementary Fig. 28 The IPCEs and stability test. IPCEs and integrated photocurrent density curves (**a**) and photocurrent density-time curves (**b**) @ 0 V_{RHE} of CCZTS/Au/MoS_2 photoelectrode after coating a CdS buffer layer and a Pt co-catalyst. Electrolyte: 0.2 M Cs_2HPO_4/CsH_2PO_4 aqueous solution.

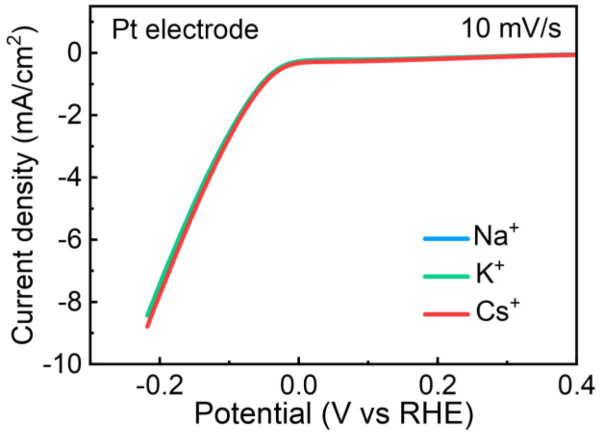

Supplementary Fig. 29 The hydrogen evolution reaction (HER) performance of Pt electrode in the different electrolytes. Electrolyte: 0.2 M Na_2HPO_4/NaH_2PO_4, K_2HPO_4/KH_2PO_4 and Cs_2HPO_4/CsH_2PO_4 aqueous solution. Scan rate: 10 mV/s.

The current-potential hydrogen evolution reaction (HER) plots of Pt electrode are almost consistent in the electrolytes with different alkali metal ions (Na^+/K^+/Cs^+), suggesting that the observed photoelectrocatalytic difference of heterojunctions does not come from the distinct HER performances of the Pt co-catalysts.

Supplementary Table 1 Sheet resistance of MoS_2, MoS_2/Pd and MoS_2/Au.

	Sheet resistance /$\Omega\cdot sq^{-1}$
MoS_2	> 15000
MoS_2/Pd	42
MoS_2/Au	32

Supplementary Table 2 The energy band levels of CCZTS and MoS_2.

	E_{CB} /V_{RHE}	E_{VB} /V_{RHE}	E_g /eV
CCZTS	-0.72 ± 0.05	0.71 ± 0.02	1.45 ± 0.03
MoS_2	-0.45 ± 0.02	0.79 ± 0.05	1.24 ± 0.03

Supplementary Table 3 The work functions and Fermi levels (E_F) of Pd and Au by ultraviolet photoelectron spectroscopy.

	Work function /eV	Fermi level /V_{RHE}
Pd	5.08	0.58
Au	5.03	0.53

Supplementary Table 4 The photocurrent density of CZTS-based photocathode in neutral aqueous solution in this study and previous literatures.

**CZTS-based Photocathode**	**Electrolyte (pH)**	**Photocurrent density ($0\ V_{RHE}$) /mA/cm^2**	**References**	**Year**
MoS_2/Au/Cd-CZTS/CdS/Pt	0.2 M Na_2HPO_4/NaH_2PO_4 (6.5)<br>0.2 M Cs_2HPO_4/CsH_2PO_4 (6.5)	-14.9<br>-16.3	This work	2023
MoS_2/CZTS/HfO_2/CdS/HfO_2/Pt	0.2 M Na_2HPO_4/NaH_2PO_4 (6.5)	-18	Huang et al[8]	2020
MoS_2/Ag-CZTS/CdS/Pt	1 M K_2HPO_4/KH_2PO_4 (7.0)	-17.7	Tay et al[9]	2020
MoS_2/Cd-CZTS/CdS/TiMo/Pt	1 M K_2HPO_4/KH_2PO_4 (7.0)	-17	Tay et al[10]	2018
MoS_2/CZTS/CdS/HfO_2/Pt	0.2 M Na_2HPO_4/NaH_2PO_4 (6.5)	-12	Huang et al[11]	2018
MoS_2/Ge-CZTS/CdS/In_2S_3/Pt	0.2 M Na_2HPO_4/NaH_2PO_4 (6.5)	-11.1	Wen et al[12]	2017
MoS_2/CZTS/CdS/TiO_2/Pt	0.5 M Na_2SO_4+0.25 M Na_2HPO_4<br>+0.25 M NaH_2PO_4 (6.85)	-11	Yang et al[13]	2016
MoS_2/CZTS/CdS/In_2S_3/Pt	0.2 M Na_2HPO_4/NaH_2PO_4 (6.5)	-9.3	Jiang et al[14]	2015

Supplementary References

1. Xiao, Y. *et al.* Band structure engineering and defect control of Ta_3N_5 for efficient photoelectrochemical water oxidation. *Nat. Catal.* **3**, 932-940 (2020).
2. Gupta, B., Melvin, A. A., Matthews, T., Dash, S. & Tyagi, A. K. TiO_2 modification by gold (Au) for photocatalytic hydrogen (H_2) production. *Renew. Sustain. Energy Rev.* **58**, 1366-1375 (2016).
3. Michaelson, H. B. The work function of the elements and its periodicity. *J. Appl. Phys.* **48**, 4729-4733 (1977).
4. Chen, M. *et al.* Faradaic junction and isoenergetic charge transfer mechanism on semiconductor/semiconductor interfaces. *Nat. Commun.* **12**, 6363 (2021).
5. Acerce, M., Voiry, D. & Chhowalla, M. Metallic 1T phase MoS_2 nanosheets as

supercapacitor electrode materials. *Nat. Nanotechnol.* **10**, 313-318 (2015).

6. Luxa, J. *et al.* Cation-Controlled Electrocatalytical Activity of Transition-Metal Disulfides. *ACS Catal.* **8**, 2774-2781 (2018).
7. Zheng, J. *et al.* High yield exfoliation of two-dimensional chalcogenides using sodium naphthalenide. *Nat. Commun.* **5**, 2995 (2014).
8. Huang, D. *et al.* 3.17% efficient Cu_2ZnSnS_4-$BiVO_4$ integrated tandem cell for standalone overall solar water splitting. *Energy Environ. Sci.* **14**, 1480-1489 (2021).
9. Tay, Y. F. *et al.* Improving the interfacial properties of CZTS photocathodes by Ag substitution. *J. Mater. Chem. A* **8**, 8862-8867 (2020).
10. Tay, Y. F. *et al.* Solution-Processed Cd-Substituted CZTS Photocathode for Efficient Solar Hydrogen Evolution from Neutral Water. *Joule* **2**, 537-548 (2018).
11. Huang, D. *et al.* Over 1% Efficient Unbiased Stable Solar Water Splitting Based on a Sprayed Cu_2ZnSnS_4 Photocathode Protected by a HfO_2 Photocorrosion-Resistant Film. *ACS Energy Lett.* **3**, 1875-1881 (2018).
12. Wen, X., Luo, W., Guan, Z., Huang, W. & Zou, Z. Boosting efficiency and stability of a Cu_2ZnSnS_4 photocathode by alloying Ge and increasing sulfur pressure simultaneously. *Nano Energy* **41**, 18-26 (2017).
13. Yang, W. *et al.* Molecular Chemistry-Controlled Hybrid Ink-Derived Efficient Cu_2ZnSnS_4 Photocathodes for Photoelectrochemical Water Splitting. *ACS Energy Lett.* **1**, 1127-1136 (2016).
14. Jiang, F. *et al.* $Pt/In_2S_3/CdS/Cu_2ZnSnS_4$ Thin Film as an Efficient and Stable Photocathode for Water Reduction under Sunlight Radiation. *J. Am. Chem. Soc.* **137**, 13691-13697 (2015).
